# Digestive Physiology of *Octopus maya* and *O. mimus*: Temporality of Digestion and Assimilation Processes

**DOI:** 10.3389/fphys.2017.00355

**Published:** 2017-05-31

**Authors:** Pedro Gallardo, Alberto Olivares, Rosario Martínez-Yáñez, Claudia Caamal-Monsreal, Pedro M. Domingues, Maite Mascaró, Ariadna Sánchez, Cristina Pascual, Carlos Rosas

**Affiliations:** ^1^Unidad Multidisciplinaria de Docencia e Investigación, Facultad de Ciencias, Universidad Nacional Autónoma de MéxicoSisal, Mexico; ^2^Departamento de Biotecnología, Facultad de Ciencias del Mar y Recursos Biológicos, Universidad de AntofagastaAntofagasta, Chile; ^3^División de Ciencias de la Vida, Departamento de Veterinaria y Zootecnia, Universidad de GuanajuatoIrapuato, Mexico; ^4^Instituto Español de Oceanografía, Centro Oceanográfico de VigoVigo, Spain

**Keywords:** *Octopus maya*, *O. mimus*, digestive physiology, digestive gland, gastric juice, digestive enzymes, assimilation process

## Abstract

Digestive physiology is one of the bottlenecks of octopus aquaculture. Although, there are successful experimentally formulated feeds, knowledge of the digestive physiology of cephalopods is fragmented, and focused mainly on *Octopus vulgaris*. Considering that the digestive physiology could vary in tropical and sub-tropical species through temperature modulations of the digestive dynamics and nutritional requirements of different organisms, the present review was focused on the digestive physiology timing of *Octopus maya* and *Octopus mimus*, two promising aquaculture species living in tropical (22–30°C) and sub-tropical (15–24°C) ecosystems, respectively. We provide a detailed description of how soluble and complex nutrients are digested, absorbed, and assimilated in these species, describing the digestive process and providing insight into how the environment can modulate the digestion and final use of nutrients for these and presumably other octopus species. To date, research on these octopus species has demonstrated that soluble protein and other nutrients flow through the digestive tract to the digestive gland in a similar manner in both species. However, differences in the use of nutrients were noted: in *O. mimus*, lipids were mobilized faster than protein, while in *O. maya*, the inverse process was observed, suggesting that lipid mobilization in species that live in relatively colder environments occurs differently to those in tropical ecosystems. Those differences are related to the particular adaptations of animals to their habitat, and indicate that this knowledge is important when formulating feed for octopus species.

## Introduction

For cephalopods, in particular for octopus, proteins are the main metabolic substrate characterized by a natural diet based mainly on crustaceans, molluscs, and fish (Alejo-Plata et al., 2009; Krstulovíc and Vrgoc, 2009; Estefanell et al., 2013). Previous studies have demonstrated the importance of crustaceans in octopus diets, showing that up to 19 crustaceans species can be found in the diet of wild *Octopus vulgaris* paralarvae (Roura et al., 2012). In recent years and due to *Octopus maya* and *Octopus mimus* reaching high market value, these species were identified as two strong candidates for marine aquaculture as they adapt well to captivity and can eat freeze-dried diets, allowing for their cultivation in ponds and tanks.

Thanks to physiological digestion research, a semi-moist paste based on squid and crab meat was recently developed as a successful diet for *O. maya* juveniles and adults (Martínez et al., 2014; Tercero-Iglesias et al., 2015). With this diet, wild females were successfully acclimated, and pre adults were grown until spawning. Although, the diet was based on the digestive capacity of juvenile octopus, it was successfully used to cultivate hatchlings until they reached 250 g body weight, as requested by the gourmet market (Rosas et al., 2014). Research in nutrition is frequently dedicated to formulating diets for cephalopod species. For wild sub-adults of *O. vulgaris*, it was recently demonstrated that dehydration of raw materials at temperatures lower than 60°C induced similar growth rates when compared to freeze dried ingredients (Rodriguez-González et al., 2015), indicating that protein characteristics are correlated with the digestive capacity of the animals. Although, there are many papers related to important ingredients (amino acids, lipids) for *O. vulgaris* feed (Cerezo-Valverde et al., 2012a,b, 2013; Estefanell et al., 2013; Querol et al., 2013; Hamdan et al., 2014; Rodriguez-González et al., 2015) there is a general lack of knowledge of the physiological processes involved during nutrient digestion in octopus species. Considering that protein digestion is a key aspect of cephalopod nutrition, the study of the process by which proteins (and other nutrients) are digested and assimilated will determine diet design (Martínez et al., 2014).

According to Boucher-Rodoni et al. (1987), cephalopod digestion can be divided into two steps: extracellular and intracellular digestion. Extracellular digestion starts in the prey, where the chyme is formed after the action of salivary gland enzymes. After the chyme is ingested by the mouth, it flows to the anterior stomach (crop) and almost simultaneously to the posterior stomach, caecum and digestive gland (DG) where it is absorbed and intracellular digestion begins. The DG plays a key role in the digestive process, where chyme nutrients are hydrolysed and transformed into acyl-glycerides, amino acids (AA), or carbohydrates (Boucaud-Camou et al., 1976; Boucher-Rodoni et al., 1987; Budelmann et al., 1997). According to Linares et al. (2015) these processes are affected not only by the type of diet, but also by habitat temperature of each species.

Some years ago, it was proposed that *O. mimus* and *O. maya* were separated from the *O. vulgaris* co-family as a result of the separation of populations provoked by the emergence of Central America. The hypothesis suggests that this new geomorphology interrupted the genetic flow between Pacific and Atlantic populations, favoring their speciation (Perez-Losada et al., 2002; Porta, 2003). Recently the thermal tolerance of *O. mimus* embryos was established in the range of 14–21°C, explaining why this species is distributed from the tropical coastal zone of northern Peru (Tumbes) to sub-tropical zone of central Chile (Bahía de San Vicente) along a natural thermal range of 15–21°C (Uriarte et al., 2012). Conversely, *O. maya* inhabits a tropical ecosystem in the Yucatán Peninsula where the benthic thermal regime is in the range of 22–26°C (Noyola et al., 2013). So although both species probably have the same evolutionary origin, it is reasonable to suppose that environmental conditions in each habitat modulate their physiology, and in consequence the manner in which each species ingest, digest, and use the nutrients obtained from food.

This paper summarizes all the steps in the digestive physiology of *O. maya* and *O. mimus* particularly regarding the digestion timing of raw nutrients: absorption, transportation, storing and use of nutrients as a source of energy. Considering that crustaceans are the primary prey of both octopus species (Leite et al., 2009) and also the most complete food type (Rosas et al., 2013), this paper summarizes the studies performed on both octopus species fed with crab meat (Martínez et al., 2011, 2014; Linares et al., 2015). Using *O. maya* and *O. mimus* as models, the results obtained to date allow us to provide a general overview of (digestive physiology in these species, that we believe can be applied to other species living in the tropics (*O. maya*) and sub-tropics (*O. mimus*). In this context, we think that this summary is valuable for the development and management of a balanced feed that would allow maintenance of octopus species in captivity under the best nutritional conditions possible.

## Starting the digestion process

Protein digestion in octopuses starts in the prey when enzyme action, mainly chymotrypsin excreted by posterior salivary glands, initiates external digestion (Boucaud-Camou and Boucher-Rodoni, 1983). As in other octopus species, chymotrypsin activity in *O. maya* and *O. mimus* was detected as the principal enzyme of salivary glands involved in external digestion, where a pre-digestion of raw flesh produces soluble proteins that form the chyme when ingested (Aguila et al., 2007; Linares et al., 2015). Forty to eighty minutes after feeding, the first chyme rapidly fills the crop, stomach and octopus caecum. Besides the obvious role as the first nutritional molecules input, we hypothesize that this first chyme could activate zymogens (acidic and alkaline enzymes) located in the gastric juice (GJ) along the digestive tract and absorption sites in the DG where those enzymes are present. This hypothesis is based on the fact that the chyme composition, besides polypeptides produced by the pre-digestion in the prey, includes active enzymes from the prey that act on the zymogens (Boucaud-Camou and Boucher-Rodoni, 1983; Hedstrom, 2002). Results obtained in *Sepioteuthis lessoniana* demonstrated that, during digestion process, zymogens were newly released after the 1 h after feeding, increasing digestive efficiency in this species. From this perspective, Martínez et al. (2011) proposed that the first pulse of AA observed in the chyme could stimulate the brush border of the acinar cells in the octopus DG (Martínez et al., 2011) increasing, as in *S. lessoniana*, the digestive efficiency in octopus species. Although, it is unknown if AA content in the chyme can stimulate the digestive cells in the DG of octopus species, in chicken intestine some AA were observed to enhance the absorption of other AA, increasing the digestive efficiency of proteins (Herzberg and Lerner, 1973). Therefore, we hypothesize that free AA in the first pulse of chyme registered in *O. maya* and *O. mimus* may facilitate nutrient absorption when more complex molecules are digested and absorbed in the second chyme pulse (Linares et al., 2015).

## Nutrient process in digestive gland

Previous experiments with *O. maya* confirmed that glucose, synthesized via the gluconeogenic pathway, is the final energetic product of protein catabolism (Martínez et al., 2011; Rosas et al., 2011; Baeza-Rojano et al., 2013). Linares et al. (2015) showed that protein catabolism and glycogen synthesis in *O. maya* and *O. mimus* followed an inverse relationship throughout the digestive process. In that study, glycogen accumulation occurred at the end of the digestive process, when the AA and polypeptides were transformed into glycogen via the gluconeogenesis pathway. Linares et al. (2015) also identified lipids as a source of energy in *O. maya*, while glycogen was the first source of energy for *O. mimus*. Such differences could be related to the type of food that was used in each experiment; *O. maya* was fed crab of the genus *Callinectes* spp. because this is the favorite prey of this octopus species. Due to its role as a molting hormone precursor, cholesterol (Chol) is highly concentrated in the crustacean DG, (Teshima, 1997; Teshima et al., 1997; Pascual et al., 2003), and therefore is readily available to octopuses. It is possible that the two Chol peaks observed in the chyme of *O. maya* were obtained first from the haemolymph and later from that stored in the crab DG (Linares et al., 2015; **Figure 2**). Results obtained by Linares et al. (2015) also suggest that both Chol and AG were mainly used as a source of metabolic energy in the DG, because those authors did not detect changes in those nutrients in the haemolymph during their study. As a key digestive organ, the DG provides digestive enzymes and stores nutrients that are used as a metabolic energy source, with glucose (*O. mimus*) and lipids (*O. maya*) being the most important (Boucaud-Camou et al., 1976; Linares et al., 2015).

As was shown by Martínez et al. (2011), DG acini of *O. maya* are characterized by columnar cellular structures of a single cell type, with heterophagosomes, heterolysosomes, and residual bodies within the cells. Like *S. officinalis*, the DG cell kinetics in *O. maya* and *O. mimus* are related to the digestive process, in which cell metabolism and cell synthesis is directly associated with chyme pulses, which in turn are related to the ingestion of food (Boucaud-Camou et al., 1976; Boucher-Rodoni et al., 1987; Perrin et al., 2004). In *O. maya, O mimus* and other octopus species, asynchrony of the digestive cells depends on the digestive moment; the same digestive cells can be observed with different roles, either synthesizing enzymes or receiving the produced chyme for absorption and assimilation (Martínez et al., 2012; Linares et al., 2015). For that reason in the early stages of cephalopod research some researchers indicated that there were different types of cells in the DG (Budelmann et al., 1997).

As in other cephalopods species, the DG of *O. maya* and *O. mimus* perform intracellular digestion and release enzymes that will be used in the extracellular digestion (Semmens, 2002). During the digestive process the chyme provokes strong changes in the DG cells due to the biochemical reactions that occur in the heterolysosomes, where nutrients are digested by powerful acidic enzymes that cause cell wear. Under this dynamic, acinar cells will be replaced after each meal, requiring energy to complete this synthesis. Previous studies carried out in our laboratory demonstrated that the DG condition of *O. maya*, measured through its enzymatic activity depends on the type of diet. Aguila et al. (2007) fed octopus varied concentrations of fish meal and fish hydrolysed protein, and found that the enzymatic activity in the DG was higher in animals fed diets that provoked lower growth than obtained in animals fed crab, which produced the highest growth rate. That study demonstrated that animal diets made with fish meal and fish hydrolysed protein have low growth rates and lower DG energy content than octopuses fed crab, suggesting that the type of food determines not only the amount of energy directed to growth, but also the energy stored in the DG that will be used to process the next meal. Those results and others obtained from experiments performed with *O. maya* indicate that the type and level of protein are the principal source of metabolic energy in the muscle, while lipids are the principal source of energy for DG intracellular metabolism (Martínez et al., 2011; Rosas et al., 2011; Baeza-Rojano et al., 2013). Similar results were observed in *O. mimus*, indicating that the biochemical pathways observed in *O. maya* could be generalized to other octopus species (Linares et al., 2015).

## Digestive enzymes

Acidic enzymes in the crop, stomach and DG were first observed in *O. vulgaris* (Morishita, 1974). Studies by Martínez et al. (2011) and Linares et al. (2015) indicate that acidic enzymes are not only present in *O. vulgaris*, but also in *O. maya* and *O. mimus*. Those enzymes were also observed in other cephalopod species such as squid and cuttlefish (Perrin et al., 2004; Cardenas-Lopez and Haard, 2005, 2009), suggesting that this type of enzyme has a key role in the digestive capacity of cephalopods. A partial characterization of the digestive enzymes in the GJ and DG of *O. maya* (Martínez et al., 2011) found that cathepsin D, which requires an acidic environment to develop maximum activity, is 18 and 72% inhibited in the GJ and DG, respectively; this indicates that as shown by Morishita (1974), acidic enzymes have an important role in the digestive process of this octopus species. However, that family of enzymes (cathepsin and pepsin) has been demonstrated to be quite sensitive to the biochemical structure of the ingested protein. In a study of myofibrillar protein susceptibility to proteases (pepsin) when meat is exposed to heating, the cooking process was observed to affect protein digestibility via a reduction of attack enzyme sites in the denatured protein (Santé-Lhoutellier et al., 2008). To test if ingredients cooked at a high temperature also affect their digestibility for octopus (via the reduction of cathepsin attack sites in cooked protein), seven experiments carried out to study the effects of several industrial cooked fish, clam and squid meal, and laboratory cooked crab meat on growth and survival of *O. maya* juveniles (Rosas et al., 2013). Results of that study showed that diets based on fresh crab paste, lyophilized crab, and squid promoted better growth rates than those observed in animals fed diets made with cooked meal. Also, the *in vitro* enzyme activity was higher in the DG of animals fed cooked ingredients than in the DG of animals fed fresh pastes, indicating that a secretagogue effect was induced in those animals as a consequence of reduced diet digestibility. Therefore, lyophilisation was considered the method that maintained native protein in octopus diets, through facilitation of cathepsin enzyme activity, and in consequence better diet digestibility (Martínez et al., 2014; Tercero-Iglesias et al., 2015). Although, the effect of the pH on the GJ and DG enzymes was only established in *O. maya* (Martínez et al., 2011), Linares et al. (2015) observed that high enzymatic activity can be obtained when the gastric juice of *O. mimus* is assayed in relatively low pH (5.5). Considering that only an 18% enzyme inhibition was observed when pepstatin A was used in *O. maya* GJ, it is possible to hypothesize that there are other cathepsins working in the acidic environments of the GJ. For example, cathepsin L activity was demonstrated in the giant squid *Dosidicus gigas* (Cardenas-Lopez and Haard, 2009), suggesting that if the DG intracellular pH is acidic in other cephalopods, then other cathepsins in addition to cathepsin D (Martínez et al., 2011) may also be present in the GJ.

**Figure 1:**
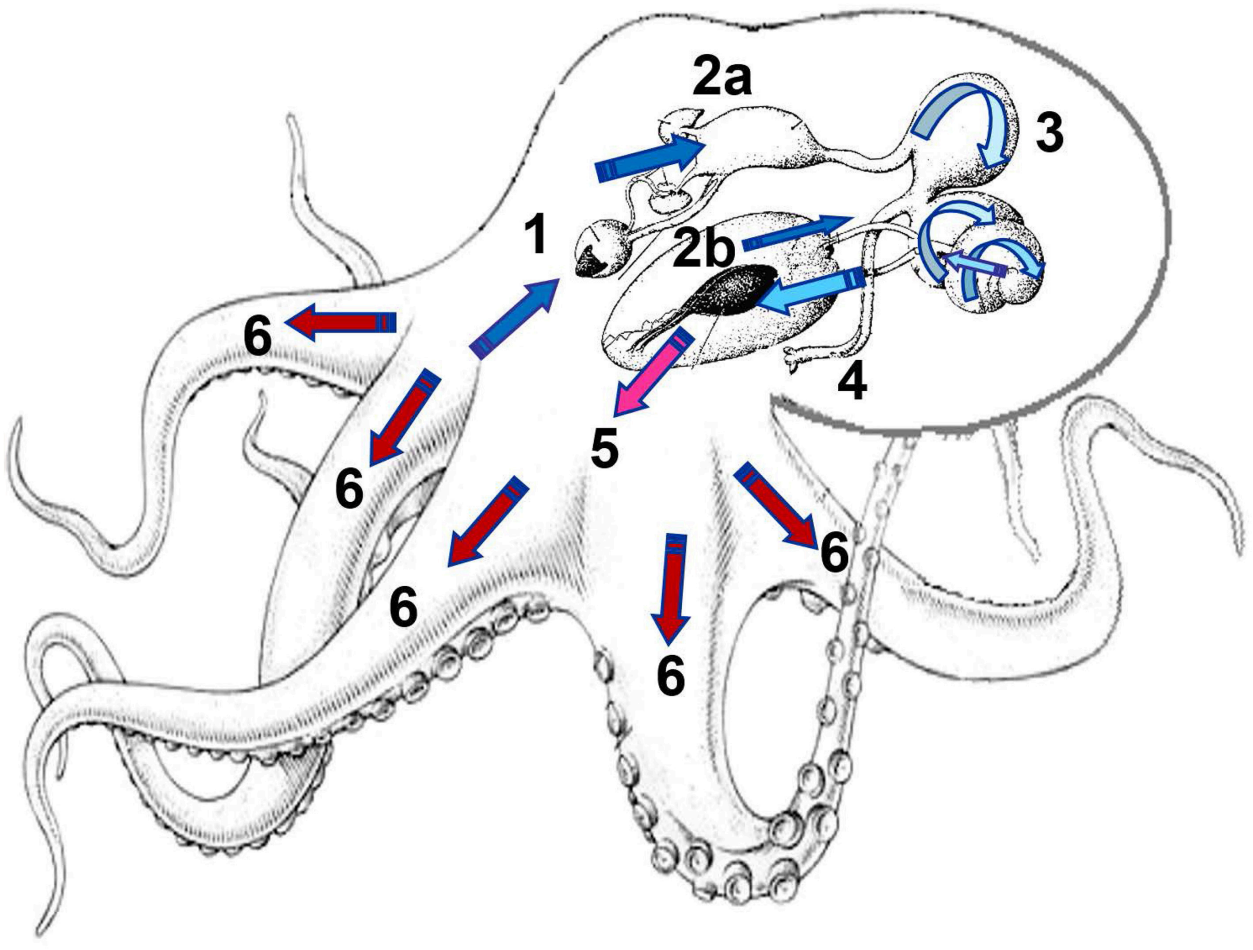
Digestive physiology of *Octopus maya* and *O. mimus*, showing the food route and the time required by the chyme to reach each part of digestive tract. 1: Mix of enzymes and soluble protein enter into the crop after the external digestion of the prey, activating zymogens in gastric juice (GJ) located along the digestive tract. 2a: Almost at the same time, soluble proteins are rapidly launched into the digestive gland (DG) activating the absorption sites, while the DG enhances its activity, absorbing nutrients and releasing enzymes in pulses (2b). Depending on the timing, alkaline and acidic enzymes act in the stomach and caecum (3). The change of color of the arrow (dark blue to clear blue) indicates changes in digestive environment, modulated by the type of enzymes involved along the digestive process changing from raw ingested food (dark blue) to assimilated and stored nutrients (red). 4: Cell debris and undigested material forming feces are sent to the anus. 5: As a result of internal digestion in the DG, amino acids (AA) and other nutrients are transported by the hemolymph to tissue to be used as a source of energy and molecules to restore tissues. In *O. maya* two peaks of AA were recorded at 20 min and 180 min after feeding (Linares et al., 2015). 6: Finally, 400–480 min after feeding nutrients are stored in tissues as muscle. Illustration: M. Linares and C. Rosas.

Results obtained until now indicate that there is synchronization between DG enzymes pulses and the GJ enzyme activity. In *O. maya* two pulses were observed (20–80 and 80–180 min), while only one pulse was noted (80–180 min) in the enzyme activity of *O. mimus*, suggesting strong differences in digestive dynamics between species (Linares et al., 2015). These differences could be due to the different environmental temperature in the habitat of each species, with more frequent enzyme release in tropical species (e.g., *O. maya*) than in subtropical or temperate species (*O. mimus*). Therefore, temperature could be regulating all the digestive activity including ingestion rate, chyme formation, intracellular digestion, and enzyme production. Digestive physiology of *O. maya* and *O. mimus* are similar in many aspects to the process described by Boucaud-Camou et al. (1976) for *O. vulgaris*. Considering the available information, a conceptual model showing the most important aspects of the *O. maya* and *O. mimus* digestive physiology was developed, which we think can be applied as a general model to other octopus species (Figure 1).

## *Octopus maya* and *O. mimus* digestive timing

The digestive physiology timing in *O. maya* and *O. mimus* is different and is probably associated with differences in habitat. *O. maya* is found in habitats where temperature fluctuates between 22 and 30°C, while *O. mimus* lives in thermal regimes that are between 14 and 22°C. Considering that type of diet and the living weight can modify the digestive timing, Linares et al. (2015) carried out their study using as a food two similar crustacean species (the blue crab *Callinectes sapidus* and *Cancer setosus*) that inhabit the adult zones of both octopus species (810 ± 116 g for *O. maya*; 1048 ± 180 g for *O. mimus*).

In that study, two digestive step processes were observed in *O. maya*: the first one was characterized by production of soluble nutrients in the prey that were rapidly ingested and absorbed, filling the digestive tract and used for muscle protein synthesis (Figure 1). After, a second slower process was identified, where more complex nutrients were obtained from muscle flesh of the prey, transformed into soluble nutrients, then transported to the DG to be catabolized and placed into muscle or stored temporarily to be used as a source of energy for the next meal (Figure 1). The digestive process of *O. mimus* was slower than in *O. maya*, showing a peak of muscle glycogen accumulation at the end of the digestive process (400 min after feeding), indicating that each species has its own timing and physiological process, related to the thermal regime in which species has evolved (Linares et al., 2015).

**Figure 2:**
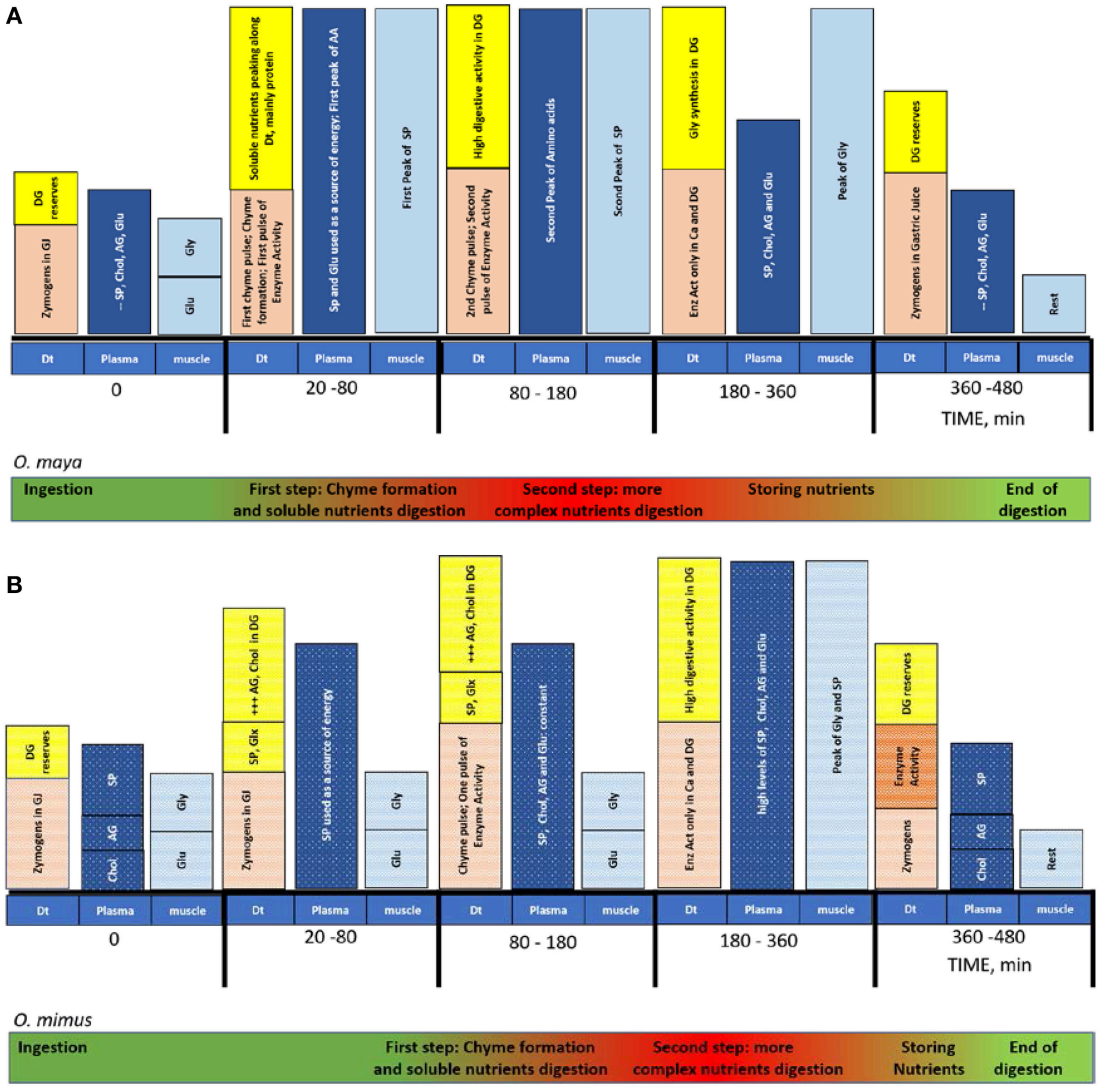
Timing of the digestive process, absorption and assimilation in adults of *O. maya* **(A)** and *O. mimus* **(B)** of the food. Before the ingestion, gastric juice (GJ) is located along the digestive tract: crop, stomach and caecum. Reserves in the DG are constant. Once the prey was offered *O. maya* took 20 min to ingest food while *O. mimus* took 140 min. While *O. maya* stored protein, *O. mimus* stored AG and Chol. The peak of the digestive process was recorded around 180 min after feeding in *O. maya* and 360 min after feeding in *O. mimus*. The end of the process was registered between 360 to 480 min in both species. Dt, digestive tract; DG, digestive gland; Ca, caecum; SP, soluble protein; Chol, cholesterol; AG, acyl glycerides; Glu, glucose; Gly, glycogen; GLx, glucose and glycogen mix; Enz, digestive enzymes; AA, amino acids. Symbol + indicates the magnitude of metabolites accumulated in DG.

Figure 2 summarizes the digestive process occurring in each section of the digestive tract of *O. maya* (Figure 2A) and *O. mimus* (Figure 2B). In that figure, all the digestive sequences that occur at the same time and along the digestive tract during digestion of each species are encapsulated. From this figure it is evident that although the general process is similar between species, there are differences in the timing of the process and the form in which DG reserves are used. In *O. mimus*, lipids were mobilized faster than proteins while in *O. maya* an inverse relationship between proteins and lipids was observed, suggesting that mobilization of lipids could be a priority in temperate octopus species (Mukhin et al., 2007). Also, it was observed that in *O. mimus* the acidic enzyme activity in the digestive tract was greater than in *O. maya*, suggesting that through modulation by low temperatures *O. mimus* could require more enzymes to digest the meal. Consequently, we think that those differences should be considered by the nutritionists who design dry foods for these octopus species. This review shows that although different octopus species have digestive systems with similar functions, each species has a unique way of digesting the food it consumes. This reflects the thousands of years of evolution that each one has experienced in different habitats, and these differences should be considered for the maintenance of healthy organisms in captive conditions.

Both *O. maya* and *O. mimus* prepare their digestive tracts for digestion. Martínez et al. (2011) and Linares et al. (2015) found digestive enzymes and zymogens in GJ along the digestive tract before meal ingestion, indicating that these GJ were secreted by the DG in preparation for the next meal. Linares et al. (2015) showed that protein, fatty acids and cholesterol in the haemolymph were the nutrients required to maintain the fasting octopus period (Figure 2A), indicating that to be prepared, octopuses not only secrete zymogens, but maintain energetic substrates in the haemolymph to be used as a source of energy between meals. This is because octopuses depend directly on the food they consume to obtain energy, as they do not have many tissue reserves to mobilize (Rosa et al., 2005), as was also shown in *O. vulgaris* (García-Garrido et al., 2011). In *O. vulgaris* 3 d of fasting was enough to induce mobilization of mantle lipids, cholesterol (Chol), acyl glycerides (AG), soluble proteins (SP), and amino acids (AA), which were used as a source of metabolic energy (García-Garrido et al., 2011). Results obtained in *O. maya* showed that this species is adapted to tolerate only short fasting periods; after 2 d of fasting a strong AA mobilization was observed (George-Zamora et al., 2011). Although, there is no information on histological changes in *O. mimus* during digestion or the use of reserves, we suspect that AA mobilization dynamics similar to those observed in *O. maya* could be present.

Both octopus species reacted immediately to offered meals, although there were differences between them. In *O. maya* adults, 20 min is enough to ingest a crab of around 100 g at 26°C (Martínez et al., 2011; Figure 2A). During this time, *O. maya* injects saliva to the prey which contains chymotrypsin and a neurotoxic fraction that causes paralysis and postural changes in the crab (Pech-Puch et al., 2016). After, the first pulse of chyme containing soluble nutrients is absorbed, initiating the digestive process. In this first chyme, high levels of soluble protein were recorded in both species along the digestive tract including the DG, where intracellular digestion begins (Figure 2A). This first pulse of chyme is the result of the chymotrypsin digestion in the prey, and is a mixture of soluble nutrients, octopus chymotrypsin, and many types of activated and inactivated prey enzymes (Figure 1; Martínez et al., 2012). Once this first pulse of chyme is ingested, it is mixed with the acidic gastric juice previously stored in the crop, stomach, and caecum resulting in the start of the acid digestion in the digestive tract. Although, a similar process was identified in *O. mimus* (Figure 2B), Linares et al. (2015) showed that adults of this species maintained at 14°C needed 80 min to ingest the food, demonstrating that temperature make more slow the digestive dynamics in these species.

As was mentioned earlier, the role of the salivary glands at the beginning of digestion process is important (Boucaud-Camou and Boucher-Rodoni, 1983; Pech-Puch et al., 2016). Enzymes from salivary glands in *O. maya* participating in the first chyme pulses are found 20 min after the meal (Pech-Puch et al., 2016). In *O. mimus* this process occurs 140 min after the meal (Linares et al., 2015), indicating again that habitat temperature of each species modulates this process (Figure 2B). Taking into consideration that the first chyme pulse could be acting as a zymogens activator (Martínez et al., 2011), timing differences in the start of digestion between species (Figure 2) could be indicating that the activation of zymogens and the timing of absorption in *O. mimus* also occurs later than in *O. maya* (Linares et al., 2015; Figure 2).

Once the digestive system in the DG is activated, heterophagosomes in the acinar cells transport energetic molecules to the haemolymph, where they are transported to muscle and other tissues (Linares et al., 2015; Figures 2A,B). Results obtained in *O. maya* and *O. mimus* provided evidence that during DG cells activation, nutrients previously stored in the DG were presumably directed to fuel the intracellular digestion (Figure 2). Reductions in DG glycogen and increments of soluble glucose in *O. maya* and *O. mimus* (Linares et al., 2015) support that idea. It is interesting to note that while soluble AG and Chol were also used as a source of energy in *O. maya* (Figure 2A), in *O. mimus* those nutrients were accumulated, indicating the importance of lipid metabolism in temperate species (Figure 2B), where lipids have a key role in maintenance of membrane fluidity in addition to their energetic role (Estefanell et al., 2013).

Differences between species can be also observed in relation to the use and destination of nutrients in the DG and other tissues (Figure 2). Haemolymph glucose levels changed significantly during the digestive process in *O. maya*, indicating that this nutrient is mobilized to support the energetic demands in different tissues of the animal (Figure 2A). The higher mobility could be necessary to satisfy the muscle energy demands in their tropical environment (Noyola et al., 2013). In contrast, glycogen was stored in *O. mimus* (Linares et al., 2015), indicating that glycogen reserves could be critical in temperate environments to maintain the DG, instead of the muscle activity observed in tropical species (Figure 2B). As in other marine invertebrates, whether they are tropical or temperate species, glycogen is an energetic product of the intermediate metabolism of non-essential AA, which is part of the muscle energy pathways (Loret, 1993) and for that reason is a key reserve molecule for the digestive process in cephalopods (Rosa et al., 2005). The role of AA in octopus energetics was demonstrated when two peaks of essential and non-essential AA were observed in *O. maya* haemolymph (Linares et al., 2015; Figure 2A). The first one was noted in conjunction with the first chyme pulse (40 min after meal) and the second one during the peak of digestive enzyme activity 140–180 min after the meal (Linares et al., 2015). This fast mobilization of AA indicates that those molecules were coupled with the digestive process, and presumably directed to tissues to be used for protein synthesis (growth) and/or glycogen synthesis (energy) (Figure 2A). In *O. maya* it was demonstrated that phenylalanine, isoleucine, alanine, glutamine, and serine are used as metabolic fuel, while histidine, arginine, and lysine are accumulated as reserves in muscle during starvation (George-Zamora et al., 2011). It is interesting to note that the observed peaks of AA in haemolymph occurred at the same time that chyme soluble protein peaked; effectively suggesting a strong mobilization of AA was the result of soluble protein catabolism (Linares et al., 2015). The role of AA as a source of energy in cephalopods was also observed in 14 other species of nektobenthic, benthic, and benthopelagic cephalopods (Rosa et al., 2005). In their study, Rosa et al. (2005), showed that proline and arginine were used as a source of energy, supporting our idea that AA are the principal energetic substrates in cephalopods via amino acid catabolism and glycogen synthesis (Rosas et al., 2002; Miliou et al., 2005). Results obtained by Linares et al. (2015) showed that glycogen peaks follow a peak of soluble protein, presumably following the digestive process:

$$\begin{array}{l} \text{Proteins in prey}>\frac{\text{Soluble proteins in the chyme}}{\text{Digestion in crop},\text{ stomach and DG}}\\ \;>\frac{\text{AA in haemolymph transported to muscle}}{\text{Intracellular enzymes in DG}}\\ \;>\frac{\text{Glucose}}{\text{Synthesis in DG and/or muscle}}\\ \;>\frac{\text{Glycogen}}{\text{Synthesis in DG and/or muscle}} \end{array}$$

Although, Linares et al. (2015) did not evaluate the haemolymph AA of *O. mimus*, considering that glycogen was also accumulated in its muscle after feeding, we conjecture that as observed in *O. maya*, the muscle glycogen in *O. mimus* was synthesized from AA, reaching its maximum value 400 min after feeding (Figure 2B).

At the end of the digestive process (480 min after feeding), a DG pH reduction was reported in *O. maya* and *O. mimus* (Martínez et al., 2011), suggesting a release of digestive enzymes in preparation for a new digestive cycle. Those enzymes could be sent to the digestive tract, where a reduction of pH was also registered (Linares et al., 2015). Reduction of enzymatic activity could also indicate that enzymes in the new gastric juice were zymogens that require the chyme to be totally activated.

Following the histological dynamics of DG in *O. maya*, Martínez et al. (2011) also observed an increment of residual body density 360 min after feeding, indicating that the feces and cellular debris removal process reached its maximum level at that time. Posteriorly, all the activity in the digestive system was reduced, with low production of residual bodies in the DG cells indicating that digestive cycle had ended (Figure 2A). At that time, nutrient reserves were accumulated in wait for the next meal (Martínez et al., 2011; Figure 2B).

As was previously stated for *O. vulgaris* by Boucaud-Camou and Boucher-Rodoni (1983), is evident the digestive physiology of *O. maya* and *O. mimus* is a fast and strongly dynamic process. In adults, this process takes around 480 min to be completed, indicating that this type of animal should be fed at least every 8 h to maintain its health in captivity (Linares et al., 2015). At a semi-pilot scale, this feed protocol has been followed for more than 5 years (Rosas et al., 2014); adults of *O. maya* were fed every 8 h using fresh scraps of marine fish or fresh crab (Caamal-Monsreal et al., 2015) or a diet formulated to stimulate spawning in laboratory conditions (Tercero-Iglesias et al., 2015). Under these conditions the number of eggs spawned was quite similar to those observed in wild spawns (Vidal et al., 2014), indicating that laboratory animals fed every 8 h reach a similar healthy condition to those on the continental shelf of the Yucatán Peninsula, where this species lives (Avila-Poveda et al., 2016; Angeles-Gonzalez et al., 2017). *O. maya* and *O. mimus* are well adapted, as are the majority of cephalopod species, to digest a high-quality animal protein diet using a mix of acidic and alkaline enzymes. This allows them to efficiently obtain the energy and molecules necessary to maintain their physiological functions according to the environment where they live, as shown for the tropical (22–30°C; *O. maya*) and temperate (14–22°C; *O. mimus*) species.

## Author contributions

CR, PG, AO, RM, and CC designed and ran the experiments in Mexico and Chile. CR, PG, PD, MM, CP, and AS, performed the laboratory analysis and processed the data. All authors contributed to write the paper.

### Conflict of interest statement

The authors declare that the research was conducted in the absence of any commercial or financial relationships that could be construed as a potential conflict of interest.
